# American Ginseng (*Panax quinquefolius*) Extracts (G1899) Ameliorate Immunosenescence via Regulation of T Cell Populations and Aging-Related Proteins in a Mouse Model Induced by D-Galactose and Tert-Butyl Hydroperoxide

**DOI:** 10.3390/cimb48030315

**Published:** 2026-03-16

**Authors:** Ji-Hye Park, Jaehoon Lee, Chang Hwan Lee, Sun Hee Hyun, Seung-Ho Lee

**Affiliations:** Laboratory of Fundamental Research, Korea Ginseng Corporation, 65, Gwacheon-daero 7-gil, Gwacheon-si 13840, Gyeonggi-do, Republic of Korea; wisdom@kgc.co.kr (J.-H.P.); 20210110@kgc.co.kr (J.L.); chlee@kgc.co.kr (C.H.L.); shhyun@kgc.co.kr (S.H.H.)

**Keywords:** American ginseng, immunosenescence, T cells, aging, Sirt1, D-galactose

## Abstract

Immunosenescence is characterized by an age-associated decline in immune function, particularly involving T-cell dysfunction, which increases susceptibility to infections and chronic diseases. This study investigated the anti-aging and immunomodulatory effects of American ginseng extract (G1899) using in vitro and in vivo models of aging. Cellular senescence was induced in HepG2 cells by D-galactose treatment, followed by exposure to G1899 (20 and 100 μg/mL). Senescence-associated markers were assessed to evaluate cellular aging. An aging mouse model was established in male C57BL/6 mice through intraperitoneal administration of D-galactose (500 mg/kg) and tert-butyl hydroperoxide (0.4 mmol/kg), and G1899 was orally administered at 400 mg/kg. Thymic immune cell subsets and aging-related protein expression were analyzed using flow cytometry and Western blotting. G1899 significantly reduced p21 expression and senescence-associated β-galactosidase activity in senescent HepG2 cells. In aging-induced mice, G1899 restored CD4^+^ and CD8^+^ T-cell populations, normalized naïve T-cell levels, and reduced anergic CD28-negative T cells. Furthermore, G1899 regulated the expression of key aging-related proteins, including FOXO1, Sirt1, p53, and CD38. These findings demonstrate that G1899 attenuates age-related immune alterations by restoring thymic T-cell homeostasis and regulating aging-associated molecular pathways.

## 1. Introduction

With advances in modern medical technology and improvements in living standards, life expectancy has rapidly increased, leading to the world’s rapid transition to an aging society [1]. Consequently, research on healthy aging has increased, and the decline in immune system function due to aging, known as immunosenescence, has emerged as a particularly important research topic in recent years [2,3]. Immunosenescence refers to the gradual decline in immune function during the aging process, and the quantitative and functional changes in T cells are known to be closely related to the development of infectious diseases, cancer, autoimmune diseases, and chronic inflammatory diseases [4,5].

Characteristic features of immunosenescence include the decrease in naïve T cells and increase in memory T cells due to thymic involution [6,7], increased T cell functional anergy [8], and decreased antigen recognition capacity [9]. Therefore, restoring or maintaining the function of aged T cells is crucial for alleviating immunosenescence and extending healthy lifespan. One strategy for this purpose is the active research on natural product-based immunomodulatory materials [10,11], and American ginseng has particularly attracted attention as various efficacies have been revealed recently [12,13].

American ginseng (*Panax quinquefolius*) belongs to the same genus as Asian ginseng (*Panax ginseng*), but it has different proportions of saponins (ginsenosides) and specific compositions of bioactive substances, reported to show differentiated efficacy in immune regulation, antioxidant, and anti-inflammatory effects [14,15]. Previous studies have revealed the efficacy of American ginseng in fatigue improvement, blood sugar control, and immune enhancement [16,17], but T cell-focused research on immunosenescence improvement is very limited [18].

This study was conducted to fill this research gap by specifically evaluating the effects of American ginseng extract (G1899) on T cell compositional changes and functional recovery in an immunosenescence model induced by D-galactose and tert-butyl hydroperoxide [19,20]. Through this, we aimed to explore the potential of G1899 as a promising natural functional material that contributes to immunosenescence suppression and immune system homeostasis maintenance [21].

## 2. Methods

### 2.1. Preparation of American Ginseng Extract (G1899)

The extraction procedure for G1899 followed the international standard production process (ISO 19610). The *Panax quinquefolius* root extract was prepared through a drying process from the Korea Ginseng Corporation (Daejeon, Republic of Korea). American ginseng was extracted using reverse osmosis water preheated to 90.0 °C ± 2.0 °C for more than 30 min as the extraction solvent. The extraction was performed in a batch-type manner, repeated five times for 8 h each at 85.0 °C ± 2.0 °C. The resulting dark-brown liquid extract was freeze-dried. The quantification of major ginsenosides in G1899 was performed experimentally using established chromatographic methods, as previously described in detail [22,23]. The final extract was reconstituted based on a moisture content of 33%, and it contained more than 50 mg/g of seven major ginsenosides and over 80 mg/g of total ginseng saponins. The specific ginsenoside profile is presented in the Results section.

### 2.2. Cell Culture and Treatment

HepG2 cells, a human hepatocellular carcinoma cell line, were obtained from American Type Culture Collection (ATCC; Manassas, VA, USA) and were cultured in Dulbecco’s Modified Eagle’s Medium (DMEM; Gibco, Waltham, MA, USA) supplemented with 10% fetal bovine serum (FBS; Gibco) and 1% penicillin-streptomycin (Gibco), maintained at 37 °C in a humidified atmosphere containing 5% CO_2_. For in vitro assays, HepG2 cells were assigned to the Normal group (Nor), the D-galactose-induced senescence group (D-galactose), and the G1899-treated group (G1899; D-galactose + G1899). To induce cellular senescence, HepG2 cells were treated with D-galactose (40 mg/mL) for 5 days, a condition commonly used to establish D-galactose–induced hepatocyte senescence models in the literature and repeatedly validated in our in-house optimization for robust and reproducible senescence induction. After senescence induction, cells were treated with G1899 (20 or 100 μg/mL) for an additional 24 h [24].

### 2.3. Cell Viability Assay

Cell viability was assessed using a Cell Counting Kit-8 (CCK-8) assay (Dojindo Molecular Technologies, Inc., Kumamoto, Japan) according to the manufacturer’s protocol. Briefly, HepG2 cells were seeded into 96-well plates at 1.0  ×  10^4^ cells per well and maintained for 24 h. For cytotoxicity screening of G1899, HepG2 cells were treated with G1899 at concentrations up to 1000 μg/mL for 24 h. Subsequently, 10 μL of CCK-8 solution was added to each well and the plates were incubated for 2 h at 37 °C. Absorbance was measured at 450 nm using a microplate reader (GloMax^®^ Discover Microplate Reader, Promega, Madison, WI, USA), and cell viability was calculated as a percentage relative to the untreated control group (*n* = 3 independent experiments with triplicate wells per experiment).

### 2.4. SA-β-Gal Staining

Senescence-associated β-galactosidase (SA-β-gal) staining was performed using a Senescence β-Galactosidase Staining Kit (Cell Signaling Technology, Danvers, MA, USA) according to the manufacturer’s protocol. This assay detects lysosomal β-galactosidase activity at pH 6.0, a widely established biomarker of cellular senescence. Stained cells were visualized under a light microscope (*n* = 3/group).

### 2.5. Immunofluorescence Confocal Microscopy

HepG2 cells were cultured on coverslips pre-coated with gelatin (SPL Life Sciences, Pocheon-si, Republic of Korea) and treated with D-galactose and G1899 as described in the previous section. After incubation, cells were fixed with 4% paraformaldehyde and permeabilized with 0.2% Triton X-100. Non-specific binding sites were blocked using 10% normal donkey serum (Abcam, Cambridge, UK), followed by overnight incubation with anti-p21 antibody (1:200 dilution, ab188224, Abcam, Cambridge, UK) diluted in the blocking solution. The samples were washed three times with PBS containing 0.1% Tween 20. And nuclear staining was performed using DAPI (Invitrogen, Carlsbad, CA, USA), while cytoskeletal staining was conducted with Alexa Fluor 555-conjugated phalloidin (Invitrogen). The expression of p21 (*n* = 3/group) was analyzed by confocal microscopy. Coverslips were mounted using ProLong™ Glass Antifade Mountant (Thermo Fisher Scientific, Waltham, MA, USA), and fluorescence images were acquired with an LSM 900 confocal microscope (Carl Zeiss, Oberkochen, Germany).

### 2.6. Animal Study Design

Seven-week-old male C57BL/6N mice were acclimated for one week prior to experimentation. The mice were housed at 25 °C ± 1 °C under a 12 h light/dark cycle. Throughout the entire experiment, the animals had free access to water and food. Mice were divided into three groups: Normal group (WT; Wild Type), Aging control group (Con; D-galactose + tBHP), and G1899-treated group (G1899; D-galactose + tBHP + G1899). Mice were injected intraperitoneally (i.p.) daily with D-galactose (500 mg/kg) in 0.9% saline for 4 weeks, with simultaneous oral administration of G1899 (400 mg/kg). To accelerate aging, tert-butyl hydroperoxide (0.4 mmol/kg, i.p.) in 0.9% saline was administered daily starting 10 days before euthanasia. Body weights, immune organ weights, and hair loss were recorded as phenotypic markers of aging. Animal experiments were approved by the Institutional Animal Care and Use Committee of the Korean Ginseng Research Institute (Daejeon, Republic of Korea) following the Guide for the Care and Use of Laboratory Animals (Approval No. KGC-2023-022).

### 2.7. Flow Cytometry Analysis of Thymocyte Populations

Thymocytes were isolated and stained using fluorochrome-conjugated antibodies against CD3-PerCP (clone 145-2C11, 1:200), CD4-FITC (clone GK1.5, 1:200), CD8-APC (clone 53-6.7, 1:200), CD44-PE (clone IM7, 1:200), CD62L-APC-Cy7 (clone MEL-14, 1:200), and CD28-PE-Cy7 (clone 37.51, 1:200) (all from BD Biosciences, San Jose, CA, USA). They were washed two times with PBS containing 2% FBS to remove excess antibody. After washing with phosphate-buffered saline (PBS), the thymocytes were resuspended in 2 mL PBS containing 2% FBS. Immune cell populations in thymocytes were analyzed using flow cytometry (BD FACS Lyric, BD Biosciences; *n* = 6 mice/group) and analyzed using FlowJo software (version 9; BD Bioscience), as presented in Table 1.

### 2.8. Western Blot Analysis

Proteins extracted from thymocytes were quantified by Bradford assay (Bio-Rad, Hercules, CA, USA). Equal amounts of protein (30 μg/lane) were separated by SDS-PAGE on 10% polyacrylamide gels and transferred to PVDF membranes (Millipore, Burlington, MA, USA). To block the membrane, it was incubated for 2 h with 5% skim milk in Tris-buffered saline with 0.1% Tween-20 (TBST), and membranes were probed with primary antibodies [anti-FOXO1 (1:1000, #2880), anti-Sirt1 (1:1000, #9475), anti-p53 (1:1000, #2527), and anti-CD38 (1:1000, #14637) (all from Cell Signaling Technology, Danvers, MA, USA)] for 24 h at 4 °C. β-actin (1:5000, sc-47778, Santa Cruz Biotechnology, Dallas, TX, USA) served as an internal control. Protein signals were developed on the membranes using ECL substrates (Thermo Fisher Scientific) and analyzed using a Fusion FX6.0 (Vilber, Collégien, France) system.

### 2.9. Statistical Analysis

Data are presented as mean ± standard deviation. Statistical analyses were performed using GraphPad Prism (GraphPad Prism 10 Software, San Diego, CA, USA). Prior to the analysis, the data were tested and confirmed to meet the assumptions of normality and homogeneity of variances. Differences among multiple groups (WT, Con, and G1899) were evaluated by one-way analysis of variance (ANOVA) followed by Tukey’s multiple-comparisons test. A *p* value < 0.05 was considered statistically significant.

## 3. Results

### 3.1. Effect of American Ginseng Extract G1899 Treatment on Aging-Related Markers in HepG2 Cells

To characterize the chemical properties of the American ginseng extract used in this study, the specific ginsenoside profile of G1899 was experimentally analyzed. The extract contains various biologically active ginsenosides, with Rb1, Re, and Rd being the most abundant (Table 2).

**Figure 1:**
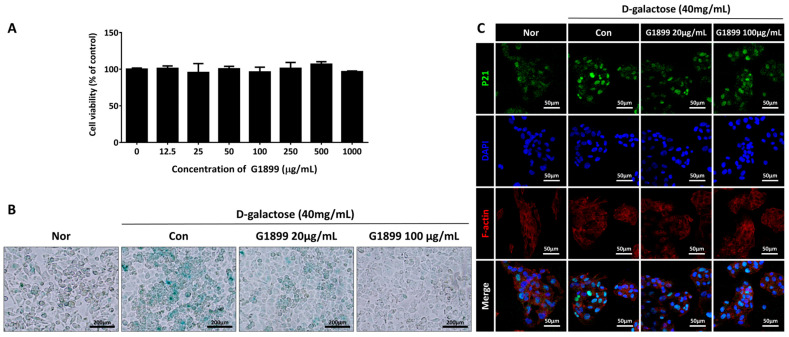
Effects of G1899 on cellular senescence markers in HepG2 cells. HepG2 cells were treated with D-galactose (D-gal; 40 mg/mL) for 5 days to induce senescence and subsequently treated with G1899 (20 or 100 μg/mL) for an additional 24 h. (**A**) Cell viability of G1899 assessed by CCK-8 assay after 24 h treatment (up to 1000 μg/mL). (**B**) SA-β-gal staining, and (**C**) Confocal microscopy analysis of p21 expression. G1899 treatment significantly reduced cellular senescence markers induced by D-gal.

Following the chemical characterization, to evaluate the cytotoxicity of G1899 in HepG2 cells, cell viability was assessed by the CCK-8 assay after 24 h of treatment with G1899 at concentrations up to 1000 μg/mL. No significant changes in cell viability were observed across the tested concentrations (Figure 1A). Based on this non-cytotoxicity screening and the subsequent anti-senescence readouts, 20 and 100 μg/mL were selected as representative low and high concentrations for subsequent mechanistic experiments in the D-galactose-induced senescence model. To assess the anti-senescence effects of G1899, HepG2 cells were treated with D-galactose (40 mg/mL) for 5 days to induce cellular senescence, followed by treatment at concentrations of 20 μg/mL and 100 μg/mL for 24 h. SA-β-gal staining showed that D-galactose treatment markedly increased senescence-associated β-galactosidase activity, whereas G1899 treatment reduced this activity in a concentration-dependent manner (Figure 1B). Additionally, p21 protein expression analysis through confocal microscopy showed decreased p21 expression in the G1899 treatment groups compared to the D-galactose treatment group (Figure 1C). These results indicate that G1899 attenuates cellular senescence induced by D-galactose in HepG2 cells, as evidenced by reduced SA-β-gal activity and decreased expression of senescence-associated proteins.

### 3.2. Effects of G1899 on Body Weight and Immune Organ Weights in D-Galactose and tBHP-Induced Aging Mouse Model

**Figure 2:**
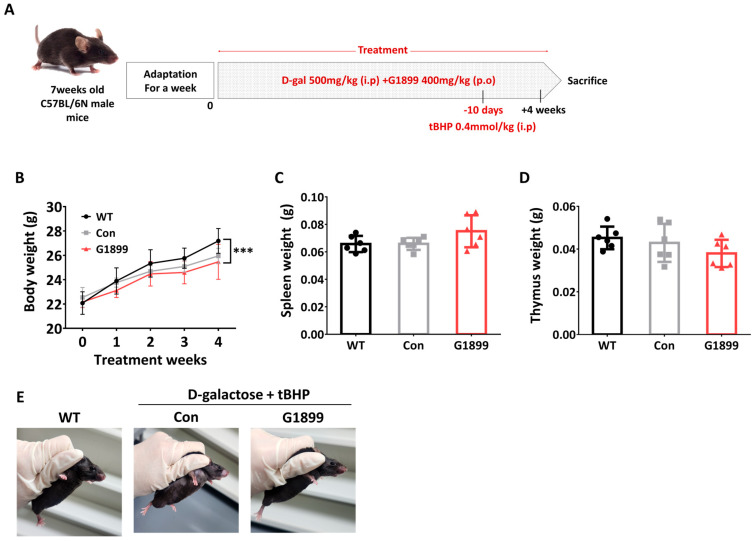
Effects of G1899 on body weight and immune organ weights in D-galactose and tert-butyl hydroperoxide (tBHP)-induced mouse model of senescence. Mice were treated with D-gal and tBHP to induce accelerated aging and simultaneously administered G1899 (400 mg/kg) (**A**). (**B**) Body weights, (**C**) spleen weights, and (**D**) thymus weights were measured. G1899 treatment did not significantly alter body or organ weights but effectively reduced external senescence phenotypes such as hair loss (**E**) (*** *p* < 0.001 vs. WT). Significance statistical analysis was performed with one-way ANOVA with post hoc Tukey test.

To evaluate the effects of G1899 in an aging-induced model, 7-week-old C57BL/6N male mice underwent a 1-week acclimation period, followed by daily treatment with D-galactose (500 mg/kg, intraperitoneal) and G1899 (400 mg/kg, oral) for 4 weeks. To accelerate aging, tert-butyl hydroperoxide (tBHP; 0.4 mmol/kg) was administered intraperitoneally daily starting 10 days before necropsy (Figure 2A). Experimental results showed no significant differences in immune organ (thymus and spleen) weights among all experimental groups (Control, D-gal, and G1899 treatment group) during the experimental period, and while there were significant differences in weight gain rates between WT and G1899 treatment groups, there were no significant weight changes due to aging induction (Figure 2B–D). However, in the external aging indicator of hair loss, the G1899 treatment group showed clear improvement effects compared to the Control group (Figure 2E). These results suggest that G1899 treatment in the accelerated aging mouse model did not alter body and immune organ weights but external indicators of aging, such as hair loss, were markedly reduced in the G1899-treated group compared with the aging control group.

### 3.3. Recovery Effects of G1899 on Thymic Immune Cell Populations in D-Galactose and tBHP-Induced Aging Mouse Model

**Figure 3:**
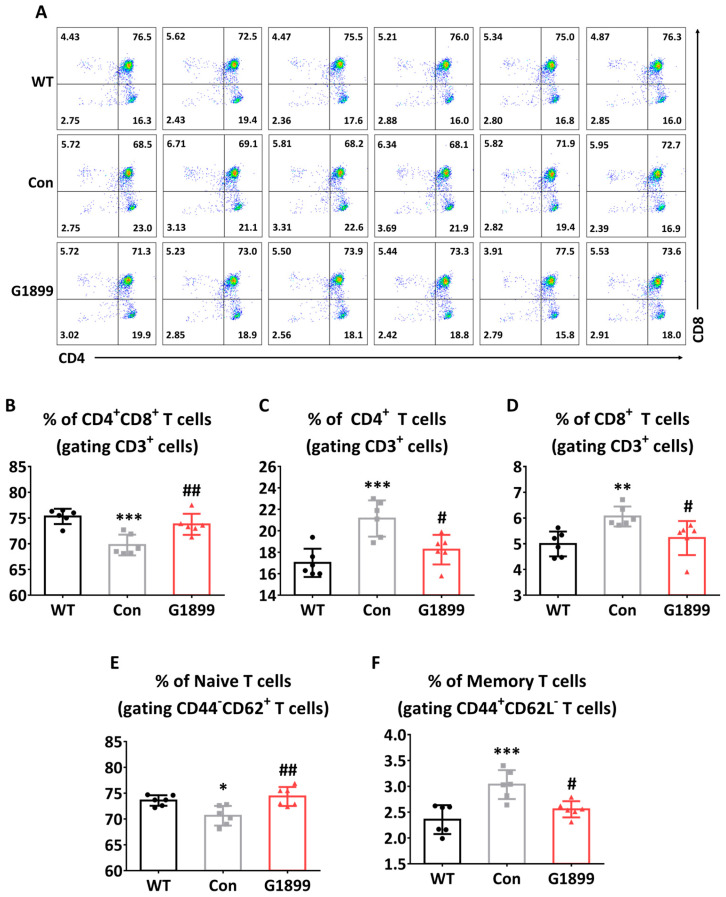
Restoration of thymic T cell populations by G1899 in a mouse model of induced senescence. Thymocytes isolated from mice were analyzed by flow cytometry. (**A**) Representative dot plots of thymocytes gated on CD4/CD8 cells, and percentage of (**B**) CD4^+^ T cells, (**C**) CD8^+^ T cells, (**D**) CD4^+^CD8^+^ T cells, (**E**) Naïve T cells, (**F**) Memory T cells were assessed. G1899 treatment significantly recovered the proportions of T cell subsets, normalizing the balance to the WT level in aging-induced mice (* *p* < 0.05, ** *p* < 0.01, *** *p* < 0.001 vs. WT; # *p* < 0.05, ## *p* < 0.01 vs. Con). Significance statistical analysis was performed with one-way ANOVA with post hoc Tukey test.

To evaluate the immune recovery effects of G1899 in the aging mouse model, thymic cells were isolated, and changes in T cell populations were evaluated through FACS analysis. Analysis results showed that CD4 and CD8 double-positive T cells decreased in the aging-induced group (Con) but recovered to levels like the WT group in the G1899 treatment group (400 mg/kg) (Figure 3B). CD4 and CD8 single-positive T cells were also confirmed to be regulated to WT levels in the G1899 treatment group (Figure 3C,D). Additionally, the ratios of naive T cells (Figure 3E) and memory T cells (Figure 3F) were normalized to WT levels in the G1899 treatment group. Statistical analysis showed significant differences between WT and Control groups, and between Control and G1899 treatment groups (* *p* < 0.05, ** *p* < 0.01, *** *p* < 0.001 vs. WT; # *p* < 0.05, ## *p* < 0.01 vs. Con). These results indicate that G1899 restored the composition of thymic T cell subsets that were altered during aging, suggesting potential immunomodulatory effects that warrant functional validation.

### 3.4. Reduction Effects of G1899 on T Cell Anergy Populations in D-Galactose and tBHP-Induced Aging Mouse Model

**Figure 4:**
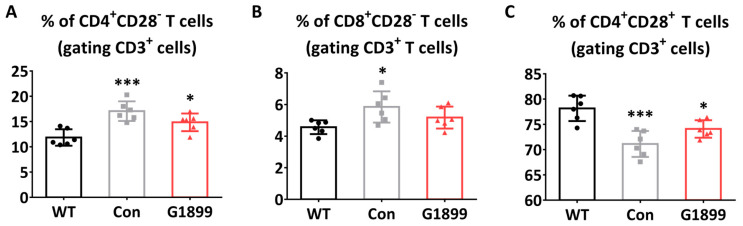
Restoration of thymic T cell populations by G1899 in a mouse model of induced senescence. Thymocytes isolated from mice were analyzed by flow cytometry. Proportions of (**A**) CD28^−^CD4^+^ T cells, (**B**) CD28^−^CD8^+^ T cells, and (**C**) CD28^+^CD4^+^ T cells were assessed. G1899 treatment significantly recovered the proportions of T cell subsets and reduced the proportion of anergic T cells, restoring levels similar to WT mice (* *p* < 0.05, *** *p* < 0.001 vs. WT). Significance statistical analysis was performed with one-way ANOVA with post hoc Tukey test.

It is known that with aging, the expression of CD28, a co-stimulatory molecule essential for T cell activation and survival, decreases, leading to an increase in CD28-negative T cells (anergic T cells). In this study, to confirm the effects of G1899 on CD28 expression changes, FACS analysis was conducted using CD3, CD4, CD8, and CD28 antibodies in thymic T cells. Analysis results showed that the proportions of CD4-positive CD28-negative and CD8-positive CD28-negative T cells were significantly increased in the aging-induced Control group compared to the WT group, but in the G1899 treatment group (400 mg/kg), the proportions were significantly restored to WT group levels (Figure 4A,B). CD4-positive CD28-positive T cells that decreased in the Control group were also confirmed to recover in the G1899 treatment group (400 mg/kg) (Figure 4C). These results suggest that G1899 administration may reduce CD28-negative T cell populations increased due to aging, improving T cell anergy.

### 3.5. Regulatory Effects of G1899 on Aging-Related Protein Expression in Thymic Cells in D-Galactose and tBHP-Induced Aging Mouse Model

**Figure 5:**
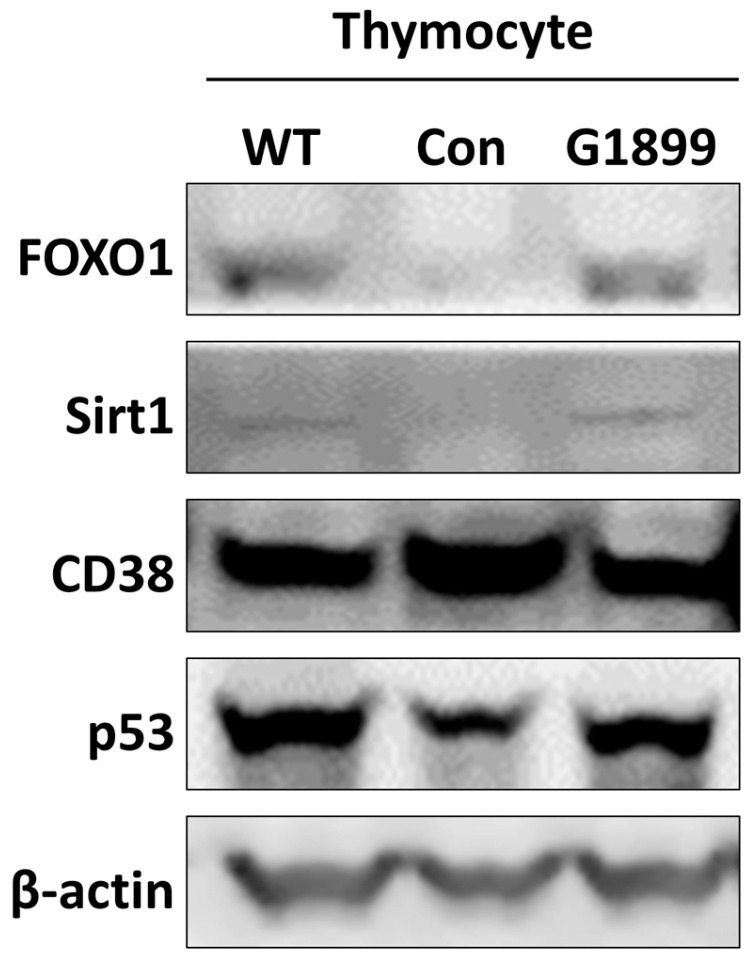
Regulation of senescence-associated protein expression in thymocytes by G1899 in aging-induced mice. Western blot analysis of thymocyte protein expressions for FOXO1, Sirt1, p53, and CD38 were performed. G1899 treatment restored the expression levels of these aging-associated proteins to near WT levels, indicating a regulatory effect of G1899 on key pathways related to immunosenescence.

The expression of aging-related proteins, including FOXO1, Sirt1, and p53, was markedly reduced in the aging control group compared with the normal group in thymic cells isolated from mice. Administration of G1899 (400 mg/kg) restored the expression of these proteins to levels comparable to those observed in the normal group. In addition, CD38 expression, which was elevated in the aging control group, was normalized following G1899 (Figure 5). These findings indicate that G1899 modulates key molecular regulators of T cell aging and suggest that the FOXO1/Sirt1 axis may be an important pathway through which G1899 exerts its protective effects against immunosenescence.

## 4. Discussion

This study demonstrates that American ginseng extract (G1899) modulates cellular senescence markers and thymic T cell subset composition in aging models [25]. The results collectively show that G1899 reduces cellular senescence markers (Figure 1), restores thymic T cell population ratios (Figure 3), normalizes CD28 expression patterns (Figure 4), and modulates key aging-related signaling pathways (Figure 5).

The initial evaluation using HepG2 cells treated with D-galactose was performed to establish a tractable in vitro model for assessing the anti-senescence potential of G1899 [26,27]. Although primary hepatocytes may more closely reflect physiological responses, HepG2 cells are widely used in aging research due to their stability, robust growth, and suitability for mechanistic assays [28]. In this context, G1899 treatment significantly reduced SA-β-galactosidase activity in a dose-dependent manner, while also decreasing p21 expression as shown by immunofluorescence [29]. These results indicate that G1899 mitigates senescence-associated changes at the cellular level, providing the mechanistic basis for further in vivo exploration [30].

In the animal model of D-galactose and tBHP-induced aging, G1899 supplementation demonstrated pronounced effects on the immune system [31]. One of the most notable findings was the restoration of thymic T cell populations (Figure 3). G1899 treatment normalized the proportions of CD4/CD8 double-positive thymocytes, single-positive CD4 and CD8 T cells, and the balance between naive and memory T cells. As thymic involution is a key driver of immunosenescence, this restoration suggests that G1899 supports thymic function and T cell development during aging [32,33]. Moreover, the reduction in CD28-negative T cells (Figure 4) highlights its role in preserving functional immune responsiveness, as CD28 expression is critical for T cell activation and its loss is a hallmark of senescent T cells [34,35].

At the molecular level, G1899 treatment restored expression of FOXO1 and Sirt1 (Figure 5), key regulators of cellular longevity and immune function [36,37]. The normalization of these proteins, along with modulation of p53 and CD38, indicates that G1899 engages multiple signaling pathways involved in DNA repair, oxidative stress response, and T cell homeostasis [38,39]. Specifically, the restoration of the FOXO1/Sirt1 axis offers a possible mechanistic link to the observed preservation of the naïve T-cell pool and the reduction in CD28-negative anergic cells. Collectively, these results support a broad anti-immunosenescence effect mediated through both cellular and molecular mechanisms [40].

In addition, the detailed component analysis of G1899 supports the chemical consistency and quality of the extract used in this study, which contributes to the reliability of the observed biological effects (Table 2). Interestingly, although G1899 did not significantly alter body weight or immune organ mass (Figure 2B–D), it alleviated external aging-associated phenotypes such as hair loss (Figure 2E). This observation suggests that its primary effects manifest at the cellular and immunological levels, rather than through gross morphological changes; yet these improvements may translate into enhanced systemic resilience against age-related decline [41,42].

From a translational perspective, the findings of this study are consistent with prior reports of American ginseng’s immunomodulatory properties but extend them by directly demonstrating its impact on immunosenescence [43,44]. The multilevel evidence presented, ranging from HepG2 cell senescence assays to in vivo thymic and molecular analyses, provides strong support for the therapeutic potential of G1899 [45]. These results suggest possible applications of G1899 as a functional food ingredient for promoting healthy immune aging [46].

However, this study has remaining limitations. First, the chemically induced aging model using D-galactose and tBHP may not fully recapitulate natural aging processes, and further validation in naturally aged models or clinical trials is required [47,48]. Second, while we confirmed the recovery of thymic T cell subsets, cytokine expression and functional immune responses associated with these changes were not evaluated [49]. Future studies should determine whether G1899 influences not only T cell subset composition but also immune functionality at the cytokine and effector levels [50]. Third, the specific bioactive constituents responsible for the effects of G1899 were not identified, and subsequent studies should focus on isolating these active compounds and elucidating their mechanisms of action. Fourth, our study employed limited dose ranges (20 and 100 μg/mL in vitro; 400 mg/kg in vivo) and did not establish comprehensive dose–response relationships. Systematic dose-optimization analyses with multiple dose levels (e.g., 100–800 mg/kg in vivo) would be valuable to determine the minimal effective dose, maximal efficacy, and therapeutic window of G1899. Future studies should include full dose–response curves with ED50/EC50 determination to inform optimal dosing strategies for potential clinical translation.

## 5. Conclusions

This study demonstrates that the American ginseng extract G1899 can attenuate aspects of immunosenescence in a D-galactose and tBHP-induced aging mouse model. Treatment with G1899 partially restored thymic T-cell subset compositions, notably by preserving the naïve T-cell population and reducing the proportion of CD28-negative anergic T cells. Furthermore, G1899 modulated the expression of aging-associated proteins, including FOXO1 and Sirt1, suggesting a potential regulatory role in maintaining T-cell homeostasis. While further functional research is necessary to confirm the full extent of these immunological benefits, these findings provide foundational evidence that G1899 may contribute to the maintenance of immune balance during the aging process.

## Figures and Tables

**Table 1:** Immunophenotypes of T lymphocytes analyzed by flow cytometry.

T Cell Subset	Marker
CD4^+^ single-positive T cells	CD3^+^CD4^+^CD8^−^
CD8^+^ single-positive T cells	CD3^+^CD4^−^CD8^+^
CD4^+^CD8^+^ double-positive T cells	CD3^+^CD4^+^CD8^+^
Naïve T cells	CD3^+^CD44^low^CD62L^high^
Memory T cells	CD3^+^CD44^high^CD62L^low^
Anergic T cells	CD3^+^CD28^−^

**Table 2:** Chemical composition and ginsenoside profile of G1899.

Category	Analyte	Content (mg/g)
Ginsenoside	Rg1	1.54
	Re	11.34
	Rf	Not detected
	Rh1	0.36
	Rg2s	2.46
	Rb1	34.36
	Rc	6.77
	Rb2	0.80
	Rd	7.94
	Rg3s	2.20
	Rg3r	0.56
	F1	0.03
	Rb3	1.51
	CO	Not detected
	Rg6	4.93
	F4	1.87
	Rh4	0.40
	F2	0.55
	CY	Not detected
	CK	Not detected
	Rk1	1.58
	Rg5	1.42
	Total ginsenoside	80.62
Ginseng polysaccharides	Total polysaccharides	62.69
Others	AF	5.21
	AFG	41.78
	Maltol	Not detected

## Data Availability

The data presented in this study are available from the corresponding author upon reasonable request.

## Abbreviations

The following abbreviations are used in this manuscript:

ANOVA	Analysis of variance
APC	Allophycocyanin
APC-Cy7	Allophycocyanin-Cyanine 7
CCK-8	Cell Counting Kit-8
DAPI	4′,6-diamidino-2-phenylindole
DMEM	Dulbecco’s Modified Eagle’s Medium
DP	Double positive
ECL	Enhanced chemiluminescence
FACS	Fluorescence-activated cell sorting
FBS	Fetal bovine serum
FITC	Fluorescein isothiocyanate
i.p.	Intraperitoneally
ISO	International Organization for Standardization
PBS	Phosphate-buffered saline
PE	Phycoerythrin
PE-Cy7	Phycoerythrin-Cyanine 7
PerCP	Peridinin chlorophyll-a protein
PVDF	Polyvinylidene difluoride
SA-β-gal	Senescence-associated β-galactosidase
SDS-PAGE	Sodium dodecyl sulfate-polyacrylamide gel electrophoresis
SP	Single positive
tBHP	tert-butyl hydroperoxide
TBST	Tris-buffered saline with Tween-20
WT	Wild type
